# Induction of ASC pyroptosis requires gasdermin D or caspase-1/11-dependent mediators and IFNβ from pyroptotic macrophages

**DOI:** 10.1038/s41419-020-2664-0

**Published:** 2020-06-18

**Authors:** Cuiping Zhang, Caiqi Zhao, Xiaoyan Chen, Rujia Tao, Sijiao Wang, Guangxun Meng, Xing Liu, Changzhou Shao, Xiao Su

**Affiliations:** 1 Department of Pulmonary Medicine, Shanghai Respiratory Research Institute, Zhongshan Hospital, Fudan University, 200032 Shanghai, China; 2 Department of Pulmonary Medicine, Xiamen Branch, Zhongshan Hospital, Fudan University, 361015 Xiamen, China; 3 Unit of Respiratory Infection and Immunity, Institut Pasteur of Shanghai, Chinese Academy of Sciences, 200031 Shanghai, China; 4 University of Chinese Academy of Sciences, 100039 Beijing, China; 5 Department of Pulmonary and Critical Care Medicine, Shanghai Pulmonary Hospital, Tongji University, 200433 Shanghai, China; 6 The Center for Microbes, Development and Health, Unit of Innate Immunity, Institut Pasteur of Shanghai, Chinese Academy of Sciences, 200031 Shanghai, China; 7 The Center for Microbes, Development and Health, Unit of Anti-Infective Immunity and Immune Diseases, Institut Pasteur of Shanghai, Chinese Academy of Sciences, 200031 Shanghai, China

**Keywords:** Cell death and immune response, Stem-cell research

## Abstract

Mesenchymal stem cells (MSCs) have been used in cell-based therapies for a variety of disorders. Some factors such as inflammatory mediators in the diseased area might damage the survival of MSCs and affect their efficacy. Pyroptosis is a form of programmed necrosis as a response for immune cells to cytosolic pathogenic stimuli. Whether MSCs develop pyroptosis under pathological stimulation, its underlying mechanism and biological significance are still unclear. Here, we found that LPS, flagellin, dsDNA, nigericin (NIG), or LPS combined with nigericin (LPS/NIG) could not induce pyroptosis in adipose-tissue-derived mesenchymal stem cells (ASCs). However, when applied the culture media collected from LPS/NIG-induced pyroptotic bone marrow-derived macrophages (BMDMs) to incubate ASCs, ASCs developed pyroptosis. Inhibition of caspases or deletion of *Caspase-1/11* in ASCs did not affect the pyroptotic macrophage media-triggered ASC pyroptosis while ablation of *Caspase-1/11* abolished BMDM pyroptosis induced by LPS/NIG. Media collected from LPS/NIG stimulated *Gsdmd*^−/−^ or *Caspase-1/11*^−/−^ BMDMs could not induce pyroptosis of ASCs. In addition, RNA-seq analysis showed that interferon (IFN)-stimulated genes were upregulated in pyroptotic ASCs. Adding IFNβ could boost LPS/NIG stimulated BMDM media-induced ASC pyroptosis. Surprisingly, the pyroptotic ASCs had a lower bactericidal ability to *P. Aeruginosa*. Taken together, induction of ASC pyroptosis requires gasdermin D or caspase-1/11-dependent mediators and IFNβ from pyroptotic macrophages.

## Introduction

Mesenchymal stem cells (MSCs) are multipotent stromal cells, which were first isolated from guinea pig bone marrow in 1967^1^. Nowadays, MSCs can be harvested from multiple tissues including adipose tissue, umbilical cord tissue, and umbilical cord blood^2^. MSCs have been tested for treating various diseases depending on their multifaceted properties: antibacterial effect, immunomodulation, trophic function, self-renewal, and multi-lineage differentiation^3^. Although 905 MSC-based clinical trials have been registered globally^4^, the clinical translation research of MSCs still faces problems. For example, MSCs might undergo autophagy or apoptosis under pathological conditions and their immunomodulatory function may be affected^5–7^. The fate of MSCs in the highly inflammatory and infectious microenvironment is under investigation. Whether microbial virulence or proinflammatory mediators in the host would induce pyroptosis of MSCs and the underlying mechanisms remain unknown.

Pyroptosis is defined as inflammasome-mediated programmed necrosis which is mainly executed by gasdermin D (GSDMD). It is regarded as an immune defense function that facilitates the disruption of pathogen-infected cells to expose intracellular bacteria to killer immune cells^8,9^. But as a form of necrosis, there are a series of proinflammatory factors produced along with pyroptosis^10^. Up to date, pyroptosis is mainly described in macrophage/dendritic cells. It is worthy of investigating whether MSCs undergo pyroptosis and if MSC pyroptosis impacts their function^11,12^.

In this study, we have observed the pyroptotic changes in adipose tissue-derived mesenchymal stem cells (ASCs) challenged by different pathogen associated molecule patterns (PAMPs) and media collected from the pyroptotic macrophages. We found that PAMPs (LPS, flagellin, dsDNA, nigericin) could not induce ASC pyroptosis directly; however, mediators from LPS/nigericin (LPS/NIG)-triggered pyroptotic macrophages induced ASC pyroptosis. More importantly, the mediator-induced ASC pyroptosis relied on the expression of GSDMD or caspase-1/11 in the pyroptotic macrophages. The pyroptotic ASCs had a reduced bactericidal capacity. For the first time, we have demonstrated that ASCs undergo pyroptosis when they meet with mediators from pyroptotic macrophages.

## Materials and methods

### Mice

Male C57BL/6J mice (8–10 weeks old) were purchased from Model Animal Research Center of Nanjing University. *Caspase-1/11*^−/−^ mice (mutate *caspase-1* and delete *caspase-11* in the 9^th^ chromosome) were kindly provided by Professor Guangxun Meng (Institut Pasteur of Shanghai, China). The mice were housed in groups with 12 h dark/light cycles and with free access to food and water. Anesthesia was induced with an intraperitoneal (ip) injection of pentobarbital sodium (50 mg/kg). Animal ethics was approved by Animal Ethics Committee of Institut Pasteur of Shanghai, Chinese Academy of Sciences, China.

### Isolation of adipose tissue-derived mesenchymal stem cells

ASCs were isolated from the adipose tissue of mice aged 6–8 weeks as described previously^13^. Briefly, adipose tissue was excised from the inguinal area and digested by collagenase type I (C1639; Sigma). Digested tissue was centrifuged and resuspended in mixed medium of Dulbecco’s modified Eagle’s medium and Nutrient Mixture F-12 (DMEM/F12; 11330032; Gibco) supplemented with 10% fetal bovine serum (FBS; 16140071; Gibco) and 1% penicillin/streptomycin (GNM-15140; GENOM). The resuspended cells were incubated at 37 °C, 5% CO_2_ until the cells were 80–90% confluence. Cells were passaged every 2–3 days by trypsinization when they reached 70–80% confluence and were used for the experiments at passages 4–6. ASCs were seeded onto 12-well plates at a density of (1–2) × 10^5^ cells per well, followed by incubation overnight.

### Isolation of bone-marrow-derived macrophages

The isolation and culture of BMDMs were performed as Pineda-Torra et al.^14^ described. Euthanized mice using standard CO_2_ asphyxiation guidelines followed by cervical dislocation, then harvested femur and tibia. Flushed the marrow cavities with RPMI 1640 medium (SH30809.01; HyClone) and collected marrow cells. After centrifuging for 5 min at 300 × *g* and eliminating erythrocytes, the remaining cells were resuspended in complete macrophage culture medium (CMCM, RPMI 1640 containing 10% FBS, 20% L929 conditioned medium, and 1% penicillin/streptomycin. Cultured cells were incubated in a humidified incubator with 5% CO_2_ at 37 °C for 6–7 days and replace CMCM at days 3 and 5. Adherent cells > 98% were judged as available macrophages by morphology.

### Cell lines and culture

C57BL/6 mice-derived wildtype (WT) and *Gsdmd*^*−*/−^ immortalized macrophage line (iBMDM) were kindly provided by Professor Xing Liu (Institut Pasteur of Shanghai, China). iBMDMs were grown in Dulbecco’s modified Eagle’s medium (DMEM; SH30243.01; HyClone) containing 10% FBS, 1% penicillin/streptomycin, 1% GlutaMAX (35050061; Gibco), and 0.04% mercaptoethanol, and passaged every 2 days. L929 and 3T3 cells, purchased from the Cell Bank of the Chinese Academy of Sciences (Shanghai, China), were maintained in DMEM, 10% FBS and 1% penicillin/streptomycin. All cells were grown at 37°C in a 5% CO_2_ incubator. The L929 cells grew to confluence and replace with fresh complete DMEM and maintained for 7 days. On day 7, the supernatants were collected as L929 conditioned medium after centrifuging at 750 × *g* for 10 min. All cell lines were recently authenticated by STR profiling and mycoplasma contamination test of all cells was negative.

### Collections of supernatants of macrophages and 3T3 cells

WT BMDMs, *Caspase-1/11*^−/−^ BMDMs, WT iBMDMs, and *Gsdmd*^−/−^ iBMDMs or 3T3 cells were seeded in six-well plates respectively and stimulated at a confluency of 80–90%. Cells were primed with 1 μg/ml LPS (L9143; sigma) for 5 h, followed by 10 μM nigericin (481990; sigma) challenge for 1 h in the serum-free DMEM/F12. Additionally, LPS-primed BMDMs were also transfected with 1 μg/ml LPS, 0.25 μg/ml ultrapure flagellin (tlrl-pafla; InvivoGen) or 2 μg/ml dsDNA (tlrl-ecdna; InvivoGen), plus 0.025% v/v FuGENE HD (E2311; Promega). After the above treatments, the supernatants were collected and centrifuged at 400 × *g* for 5 min before using to incubating ASCs.

### Stimulation of adipose tissue-derived mesenchymal stem cells

ASCs were primed with 0.5 μg/ml LPS for 4 h and then transfected with 1 μg/ml LPS, 0.25 μg/ml ultrapure flagellin, 2 μg/ml dsDNA, or 10 μM nigericin, plus 0.025% v/v FuGENE HD. In addition, LPS-primed ASCs were also incubated with the specific supernatants collected from macrophages or 3T3 cells for 2 h. The caspase-1 inhibitor VX765 (HY-13205; MedChemExpress) or caspase pan-inhibitor z-VAD-FMK (HY-16658; MedChemExpress) pretreated ASCs before priming of LPS. In the IFNβ (B276240; BioLegend) treatment assay, ASCs were primed with LPS (0.5 μg/ml) and IFNβ (0.4 ng/ml) for 4 h followed by related stimulation.

### Bacterial culture and infection

*Pseudomonas aeruginosa* (GFP-PAO1, a gift from Professor Yuanlin Song, Zhongshan Hospital, Fudan University) was incubated in Luria-Bertani (LB) broth overnight under aerobic conditions at 37°C and were subcultured (1:10) for 3 h in fresh LB broth to attain the mid-log growth phase. After the treatment of SC3 (control supernatant collected from 3T3 cells), SLN3 (supernatant collected from LPS/NIG-treated 3T3 cells), SCB (control supernatant collected from BMDMs), and SLNB (supernatant collected from LPS/NIG-treated BMDMs), ASCs were infected with GFP-PAO1 for 2 h at a MOI of 1 in fresh medium. Then, the culture medium was harvested and the number of viable bacteria was determined by the plate count method. In the meantime, the cells were washed thrice and incubated in DMEM with 200 μg/ml of gentamicin sulfate for 2 h to eliminate the membrane-bound bacteria. Subsequently, the intracellular fluorescence of ASCs was measured by a FACScan flow cytometer (BD fortessa, USA), which represented the number of intracellular bacteria^15^.

### RNA sequencing

LPS-primed ASCs exposed to SC3, SLN3, SCB, and SLNB (three biological replicates) were harvested and the total RNA was extracted using TRIzol reagent (Invitrogen, Carlsbad, CA). After quality test, the total RNA of each sample was sequenced using the BGISEQ-500 platform.

### Lactate dehydrogenase release assay

The lactate dehydrogenase (LDH) released from cells into culture supernatants was measured using the LDH Cytotoxicity Assay Kit (88953; Thermo Scientific) according to the manufacturer’s protocol. The percentage of LDH release was calculated as follows: % LDH release = (compound-treated LDH activity-spontaneous LDH activity) (maximum LDH activity- spontaneous LDH activity)^−1^ × 100.

### Enzyme-linked immunosorbent assay

Concentrations of IL-1β in culture supernatants were measured by Mouse IL-1β enzyme-linked immunosorbent assay (ELISA) kit (DY008/DY401-05; R&D Systems) according to the manufacturer’s instructions. Active caspase-1 in culture medium was detected using an ELISA kit from Keshun Biotech.

### Western blot analysis

For western blot, cells were lysed by cell lysis buffer (P0013; Beyotime) and cell culture supernatant (serum free) was precipitated by methanol and chloroform. The acquired protein samples were boiled for 10 min at 100 °C before gel electrophoresis. A 5200 Multi Chemiluminescence Imaging System (Tanon) was used to detect the protein bands on blots with Super ECL Detection Reagent (36208ES; Yeasen). The primary antibody used in western blot analysis were as follows: Anti-tubulin (11224-1-AP; proteintech); anti-caspase-11 (14340s; CST); anti-Caspase-1 (22915-1-AP; Proteintech); anti-GSDMD (ab209845; Abcam); anti-IL-1β (ab9722; Abcam).

### Quantitative real-time PCR

Total RNA was extracted by TRIzol reagent and reverse transcribed into cDNA with FastKing RT Kit (KR116-02; Tiangen), followed by quantitative RT-PCR analysis using SYBR Green master mix (11203ES08; Yeasen) on AB RT-PCR 384 System (USA). The primer sequences were as follows: *IFNβ* (forward) 5′-GCC TTT GCC ATC CAA GAG ATG C-3′ and (reverse) 5′-ACA CTG TCT GCT GGT GGA GTT C-3′; *IFNγ* (forward) 5′-GCA TCT TGG CTT TGC AGC T-3′; and (reverse: 5′-CCT TTT TCG CCT TGC TGT TG-3′).

### Statistics analysis

The experiments shown in this study were replicated at least 3 times in the laboratory. Data analyses were performed using GraphPad Prism software version 7.0 and FlowJo (FlowJo, Ashland, OR, USA). All quantitative data were shown as mean ± SD. The statistical significance of the differences between various treatments was determined by a two-tailed *t* test for two groups or ANOVA for three or more groups. The significance was set as *p < 0.05; **p < 0.01; ***p < 0.001; ****p < 0.0001.

## Results

### LPS, flagellin, dsDNA, or nigericin could not induce ASC pyroptosis

**Fig. 1:**
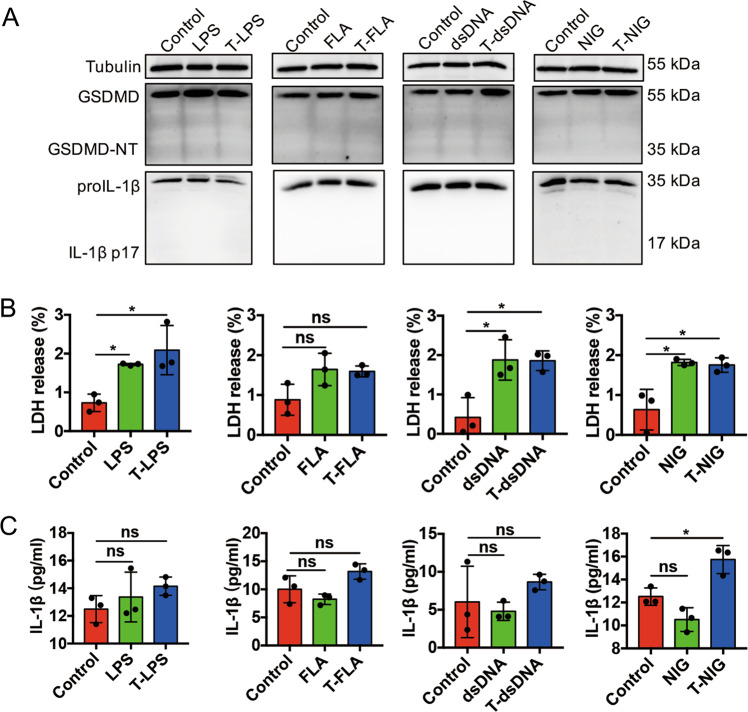
LPS, flagellin, dsDNA or nigericin cannot induce ASC pyroptosis. ASCs were primed with 0.5 μg/ml LPS for 4 h, prior to challenging with 1 μg/ml LPS, 0.25 μg/ml flagellin, 2 μg/ml dsDNA, and 10 μM nigericin respectively, with or without 0.025% v/v FuGENE HD. Pyroptosis was detected by immunoblot analysis (**a**) of GSDMD-NT and mature IL-1β in ASCs, ELISA detection of mature IL-1β (**b**) and LDH release (**c**) in cell supernatant. *n* = 3. Tubulin was used as an internal control for immunoblot analysis. Data are representative of at least three independent experiments. Statistical significance between the control group and treated groups was determined using one-way analysis of variance. Data are shown as mean ± SD. ns: *p* > 0.05, *p < 0.05. LPS lipopolysaccharide, FLA flagellin, NIG nigericin, T transfect, ASCs adipose tissue-derived mesenchymal stem cells, GSDMD gasdermin D, GSDMD-NT N-terminal gasdermin D, ELISA enzyme-linked immunosorbent assay, LDH lactate dehydrogenase.

We first addressed the question of whether ASCs sensed PAMPs in the cytoplasm and initiated pyroptosis. To increase the expression of GSDMD and IL-1β, we primed ASCs with LPS for 4 h^16^. GSDMD-mediated pyroptosis requires cytosolic inflammasome, which mainly includes caspase-11, NLRC4, AIM2, and NLRP3 inflammasomes, responding to intracellular microbial signals^17^. We applied transfection reagent to transfect LPS (caspase-11), flagellin (NLRC4), dsDNA (AIM2), and nigericin (NLRP3) into ASCs in order to activate corresponding inflammasomes. However, the immunoblotting analyses demonstrated that GSDMD and IL-1β could not be cleaved in ASCs under those treatments (Fig. 1a). There was a slight increase of LDH but no detectable mature IL-1β in the supernatant of culture media (Fig. 1b, c) no matter whether transfection of stimuli was performed. These findings indicated that LPS, flagellin, dsDNA, and nigericin could not induce ASC pyroptosis by means of activating inflammasomes.

### Media collected from LPS/NIG-triggered pyroptotic macrophages induces pyroptosis of ASCs

**Fig. 2:**
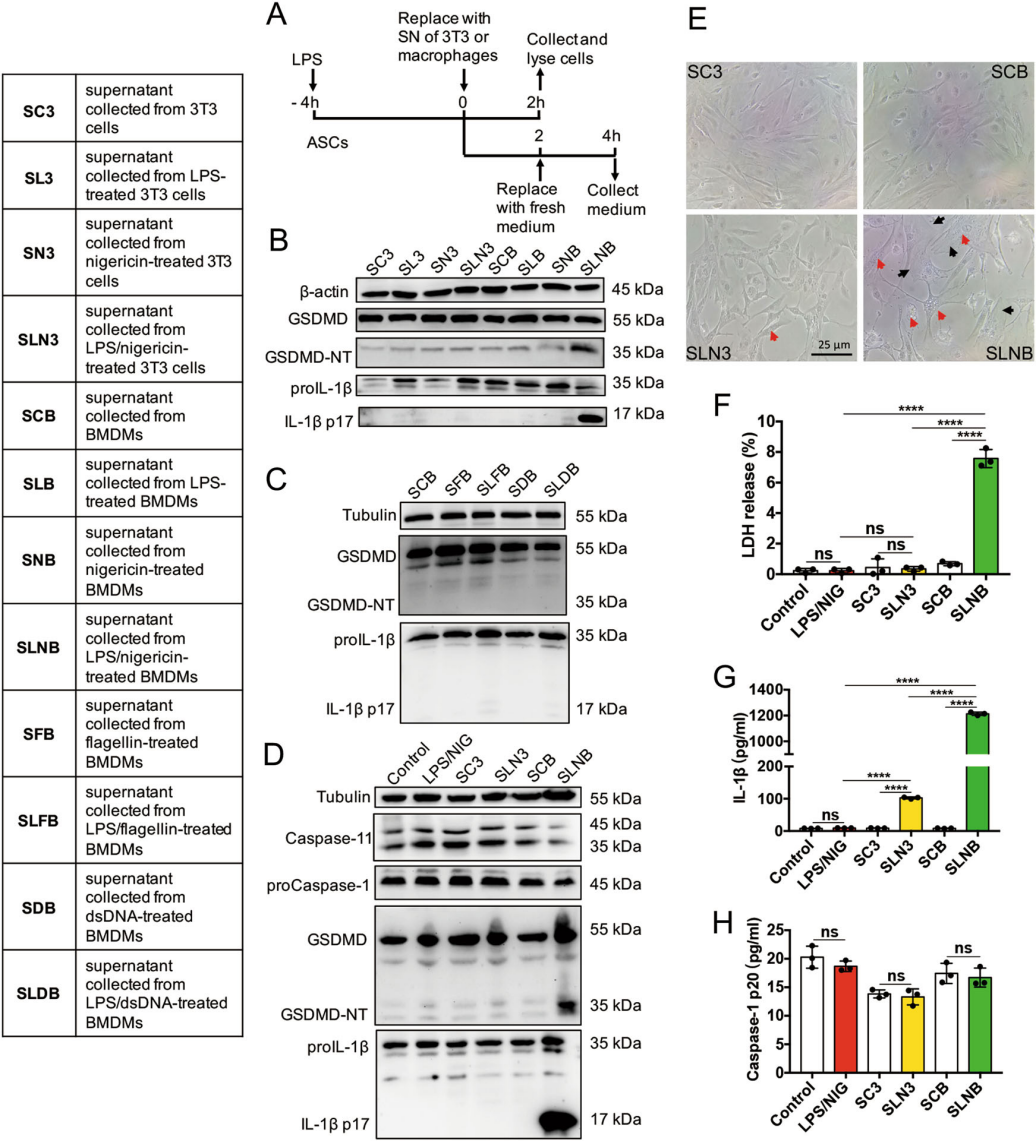
Supernatant collected from LPS/NIG-treated BMDMs induces pyroptosis of ASCs. The table showed the different supernatants collected from BMDMs or 3T3 cells. **a** LPS-primed ASCs were treated with supernatants of 3T3 cells and BMDMs for 2 h. After treating with related supernatants, the cells were collected and lysed for immunoblot analysis or replaced the supernatant with fresh media for another 2 h. At the end of experiment, the medium was harvested for detecting LDH, mature IL-1β, and caspase-1 p20 release of ASCs. Immunoblot analysis of caspase-11, proCaspase-1, GSDMD, and IL-1β in cell lysates of ASCs. **b**–**d** immunoblot analysis of caspase-11, proCaspase-1, and GSDMD, IL-1β in cell lysates of ASCs after treating with indicated supernatant. Data are representative of at least three independent experiments. LPS-primed ASCs were treated with 1 μg/ml LPS plus 10 μM nigericin or the indicated supernatants of 3T3 cells and BMDMs for 2 h. After the above treatments, collected the cells for immunoblot analysis or replaced the supernatant with fresh media for another 2 h. **e** Representative microscopic photographs of ASCs treated with SC3, SCB, SLN3, and SLNB respectively. As stated in **a**, photographs were taken by at 2 h after treatment. Objective magnification: ×40, bar 25 μm. Red arrows indicated vacuoles; black arrows indicated cell swelling. **f**–**h** As stated in **a**, the medium was harvested for detecting LDH (**f**), mature IL-1β (**g**), and caspase-1 p20 (**h**) release levels of ASCs. Data are representative of at least three independent experiments. *n* = 3. Statistical significance was determined using one-way analysis of variance. Data are shown as mean ± SD. ns: *p* > 0.05, ****p < 0.0001. LPS lipopolysaccharide, NIG nigericin, ASCs adipose tissue-derived mesenchymal stem cells, BMDMs Bone-marrow-derived macrophages, SN supernatant, GSDMD gasdermin D, GSDMD-NT N-terminal gasdermin D, LDH lactate dehydrogenase.

According to Supplemental Fig. 1a–c, we confirmed that LPS/NIG failed to triggered pyroptosis of ASCs or 3T3 cells but fiercely irritated BMDMs’ pyroptosis, which manifested as the production of GSDMD-NT and IL-1β p17 in the cells, as well as the increases of IL-1β p17 and LDH levels in the supernatant^11^. However, as shown in Fig. 2a, we primed the ASCs with LPS for 4 h and refreshed the supernatant collected from BMDMs or 3T3 cells (as a control). After 2 h incubation with specific supernatant, ASCs were collected to perform western blot or replace the supernatant with fresh media to further incubate for 2 h. The media was used to measure LDH, IL-1β, and caspase-1 p20. We found that SLNB-stimulated LPS-primed ASCs underwent pyroptosis, manifested by the production of GSDMD-NT and active IL-1β in cell lysates of ASCs (Fig. 2b, d). We also applied the supernatant collected from flagellin, dsDNA, LPS/flagellin, or LPS/dsDNA-stimulated BMDMs to LPS-primed ASCs and found that these supernatants could not induce pyroptosis in ASCs (Fig. 2c). Under microscope, we found that SC3-treated and SCB-treated ASCs were cord-shaped, there no significant difference in cell shape and intracellular contents. The cell body of SLN3-treated ASCs was slighted enlarged, and a small number of vacuoles were formed intracellularly (red arrow indicated). In the SLNB-stimulated ASCs, the cell body was significantly swollen (black arrow), and a large number of vacuoles (red arrow) were presented in the peri-nuclear region (Fig. 2e), suggesting that there was pyroptosis in SLNB-treated ASCs. LDH and IL-1β levels in the media collected from SLNB-stimulated LPS-primed ASCs were increased compared to the other group (Fig. 2f, g). Caspase-1 p20 in the media collected from SLNB-stimulated LPS-primed ASCs was not changed (Fig. 2h), suggesting that ASCs were not able to release active caspase-1.

### Inhibition or deletion of caspase-1/11 does not affect SLNB-triggered pyroptosis in ASCs

**Fig. 3:**
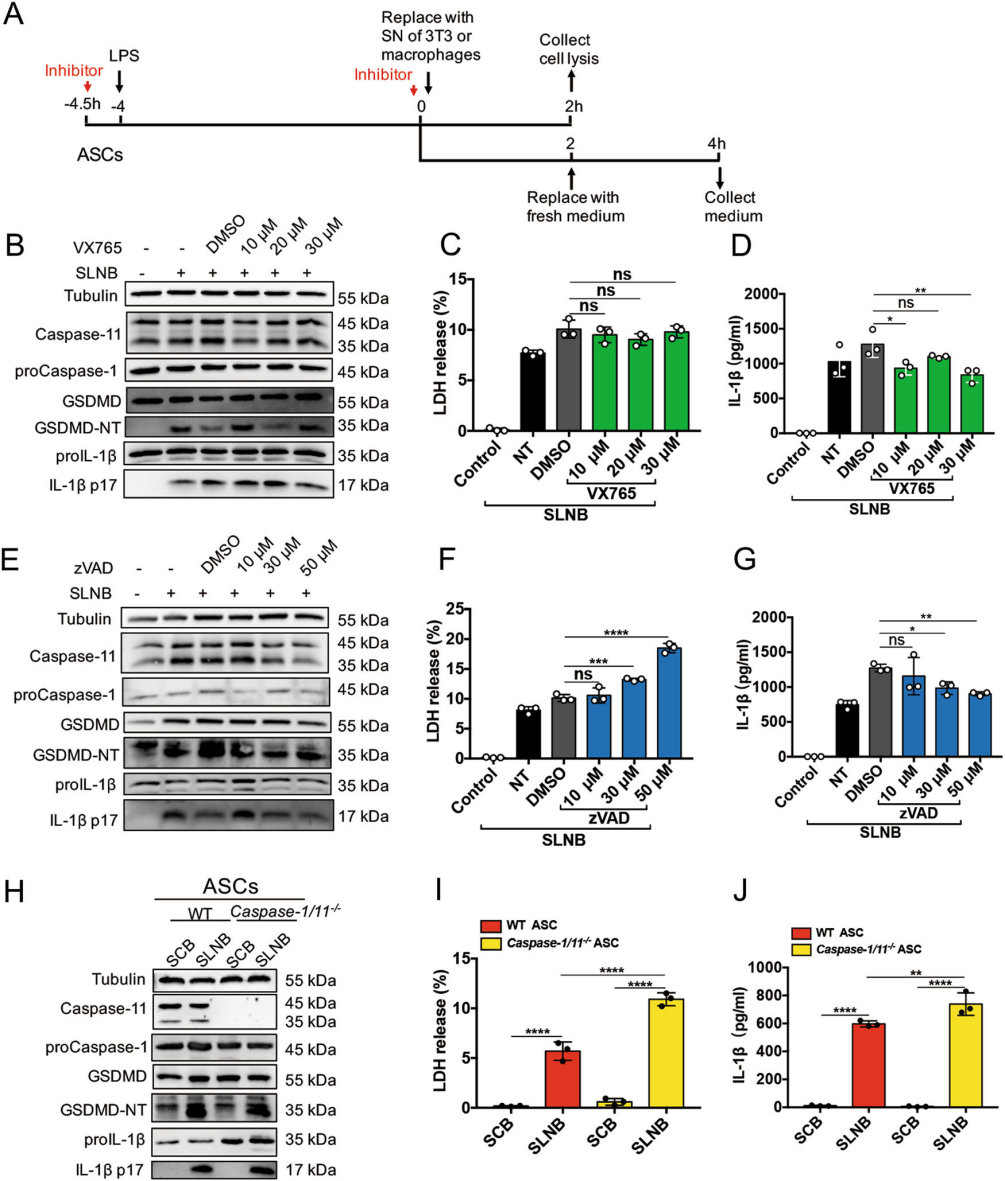
The cleavage of GSDMD and proIL-1β in ASCs do not rely on the caspases of ASCs. **a** ASCs were pretreated with VX765 or zVAD of indicated concentrations for 0.5 h before being primed with 0.5 μg/ml LPS for 4 h. Then ASCs were stimulated by SLNB mixed with VX765 or zVAD of indicated concentrations for 2 h. After the above treatments, replace the supernatant with fresh media for another 2 h-incubation or collect cells for immunoblot analyses (**b**, **e**) of indicated proteins. Data are representative of at least three independent experiments. **c**, **f** LDH release level of ASCs in the medium of **a**. *n* = 3. **d**, **g** ELISA detection of mature IL-1β in the media of **a**. *n* = 3. **h**–**j** LPS-primed WT and *caspase-1/11*^*−/−*^ ASCs were treated with SCB and SLNB for 2 h. Then replace the supernatant with fresh media for another 2-h-incubation or collect cells for immunoblot analyses (out of 3 performed) **h** of indicated proteins. LDH (**i**) and mature IL-1β (**j**) were detected in the medium. *n* = 3. Statistical significance was determined using one-way analysis of variance. Data are shown as mean ± SD. ns: *p* > 0.05, *p < 0.05, **p < 0.01, ***p < 0.001, ****p < 0.0001. zVAD z-VAD-FMK, LPS lipopolysaccharide, ASCs adipose tissue-derived mesenchymal stem cells, BMDMs Bone-marrow-derived macrophages, SN supernatant, GSDMD gasdermin D, GSDMD-NT N-terminal gasdermin D, SLNB supernatant collected from LPS/NIG-treated BMDMs, NT no treatment of inhibitor, DMSO dimethyl sulphoxide, LDH lactate dehydrogenase, ELISA enzyme-linked immunosorbent assay.

We found that caspase-1 inhibitor VX765 could inhibit LPS/NIG triggered pyroptosis in BMDMs by disrupting cleavage of GSDMD, proIL-1β (Supplemental Fig. 1d) and preventing LDH release (Supplemental Fig. 1e). Next, we examined the roles of caspase-1 or caspase-11 in pyroptosis of ASCs stimulated with SLNB. Following the protocol listed in Fig. 3a, we pretreated the ASCs with either vehicle or caspase-1 inhibitor VX765 or caspase pan-inhibitor zVAD and then incubated them with SLNB. We found that VX765 and zVAD did not affect the cleavage of GSDMD and proIL-1β in ASCs (Fig. 3b, e). In VX765/SLNB treated ASCs, LDH in the supernatant of culture media did not change, but IL-1β was lower compared to vehicle/SLNB-treated ASCs (Fig. 3c, d). In zVAD/SLNB treated ASCs, LDH in the supernatant of culture media was increased, but IL-1β was decreased compared to vehicle/SLNB-treated ASCs (Fig. 3f, g).

Furthermore, we isolated and cultured WT and *Caspase-1/11*^−/−^ ASCs to observe whether deficiency of caspase-1/11 affects SLNB to induce ASC pyroptosis. We applied SCB and SLNB to incubate WT and *Caspase-1/11*^−/−^ ASCs respectively. In the *Caspase-1/11*^−/−^ ASC lysates, caspase-11 expression was absent; however, proCaspase-1 was present but non-functional since the caspase-1 gene was mutated. After treatment with SLNB, GSDMD-NT and IL-1β p17 were produced in both WT and *Caspase-1/11*^−/−^ ASCs (Fig. 3h). In the supernatant of SLNB-challenged WT ASCs, LDH and IL-1β were increased compared to SCB-challenged WT ASCs. However, LDH and IL-1β were further increased in the supernatant of SLNB-challenged *Caspase-1/11*^−/−^ compared to SLNB-challenged WT ASCs (Fig.3i, j). These results of *Caspase-1/11*^−/−^ ASCs showed a striking contrast with the condition of *Caspase-1/11*^−/−^ BMDMs that deficiency of caspase-1/11 abolished pyroptosis in BMDMs (Supplemental Fig. 2a–c).

Taken together, these findings suggested that ASC caspases do not play a role in the SLNB-triggered pyroptosis.

### Media collected from LPS/NIG-triggered *Caspase-1/11*^−/−^ or *Gsdmd*^−/−^ macrophages is not able to induce pyroptosis in ASCs

**Fig. 4:**
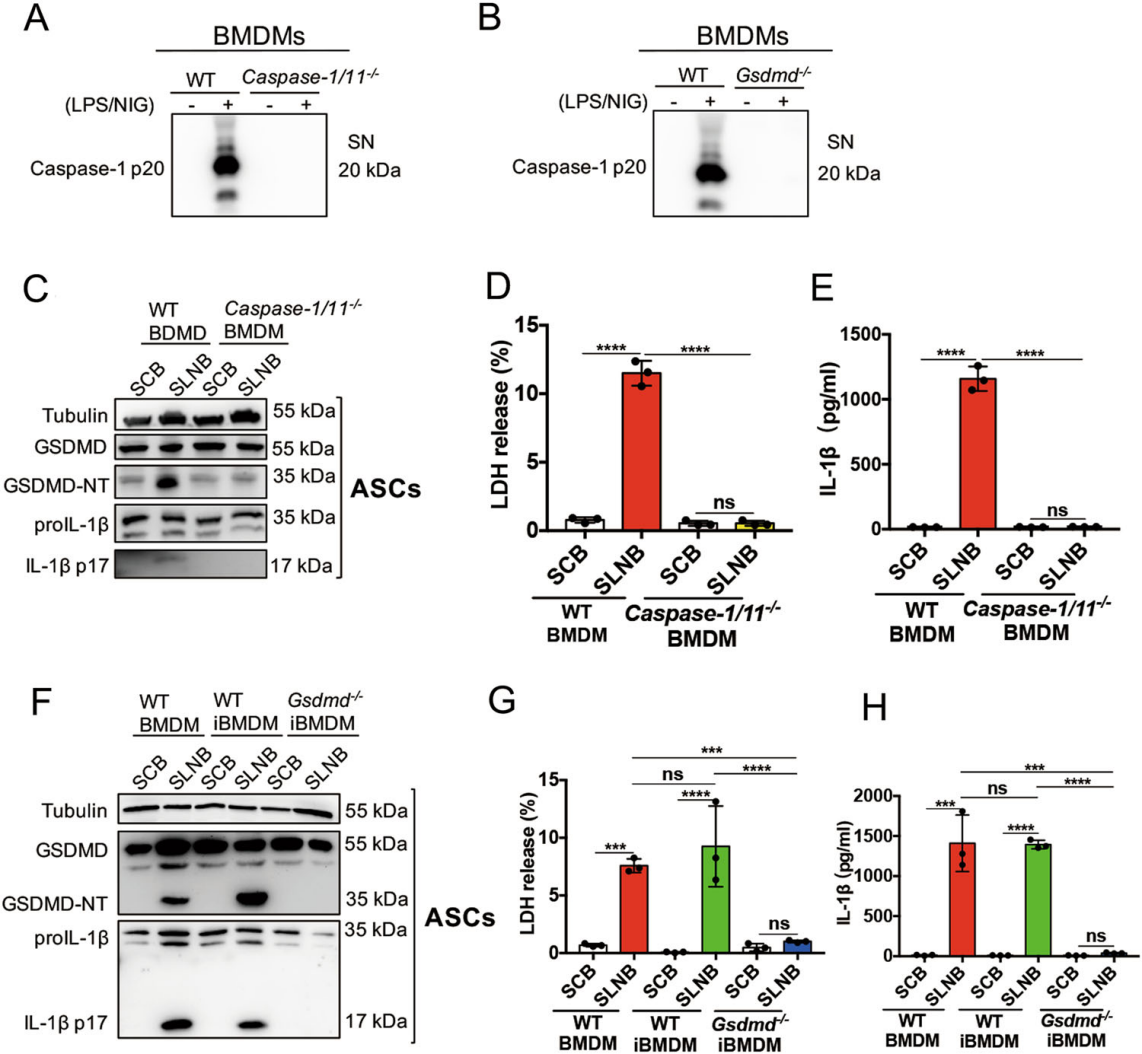
The supernatants from LPS/NIG-treated *caspase-1/11*^*−/−*^ or *Gsdmd*^*−/−*^ macrophages cannot cause pyroptosis of ASCs. **a**, **b** WT BMDMs, *caspase-1/11*^*−/−*^ BMDMs, WT iBMDMs, and *Gsdmd*^*−/−*^ iBMDMs were stimulated with 1 μg/ml LPS for 5 h prior to challenging with 10 μM nigericin for 1 h. The supernatants were collected for detecting caspase-1 p20 by immunoblotting analysis (**a**, **b**). Data are representative of at least three independent experiments. **c**–**h** Treat LPS-primed ASCs with the above supernatants for 2 h. After the treatments of related supernatants, collect ASCs for immunoblot analysis (out of at least three performed) of GSDMD and IL1β (**c**, **f**) or replace the supernatant with fresh media for another 2 h. Harvest the medium for evaluating LDH (**d**, **g**) and mature IL-1β release levels (**e**, **h**) of ASCs. *n* = 3. Statistical significance between the control group and treated groups was determined using one-way analysis of variance. Data are shown as mean ± SD. ns: *p* > 0.05, ***p < 0.001, ****p < 0.0001. LPS lipopolysaccharide, NIG nigericin, ASCs adipose tissue-derived mesenchymal stem cells, BMDMs Bone marrow-derived macrophages, iBMDMs immortalized bone marrow-derived macrophages, WT wildtype, GSDMD gasdermin D, GSDMD-NT N-terminal gasdermin D, SN supernatant, SCB supernatant collected from BMDMs, SLNB supernatant collected from LPS/NIG-treated BMDMs, LDH lactate dehydrogenase.

Consistent with previous studies^11,18^, *Caspase-1/11*^*−/−*^ and *Gsdmd*^−/−^ macrophages could not undergo pyroptosis (Supplemental Fig. 2a–f). We collected SLNB from *Caspase-1/11*^*−/−*^ BMDMs and *Gsdmd*^−/−^ iBMDMs to incubate LPS-primed ASCs. By western blot, we confirmed that caspase-1 p20 was undetectable in the SLNB from *Caspase-1/11*^*−/−*^ BMDMs and *Gsdmd*^−/−^ iBMDMs (Fig. 4a, b). When applied the SLNB from WT BMDMs to LPS-primed ASCs, high levels of GSDMD-NT and IL-1β p17 were detected by western blot (Fig. 4c, f). When applied SLNB from *Caspase-1/11*^*−/−*^ BMDMs or *Gsdmd*^−/−^ iBMDMs to LPS-primed ASCs, GSDMD-NT and IL-1β p17 were undetectable (Fig. 4c, f). We measured LDH and IL-1β in the WT BMDM-SLNB treated ASCs, LDH and IL-1β levels were increased (Fig. 4d, e, g, h). LDH and IL-1β levels in the supernatant collected from *Caspase-1/11*^*−/*−^ BMDM or *Gsdmd*^−/−^ iBMDM-SLNB treated ASCs were almost undetectable (Fig. 4d, e, g, h). These findings indicate that macrophage-released GSDMD and caspase-1/11-dependent mediators are inducers of ASC pyroptosis during infection and inflammation.

### Upregulation of interferon and NOD-like receptor signaling in SLNB-stimulated ASCs

**Fig. 5:**
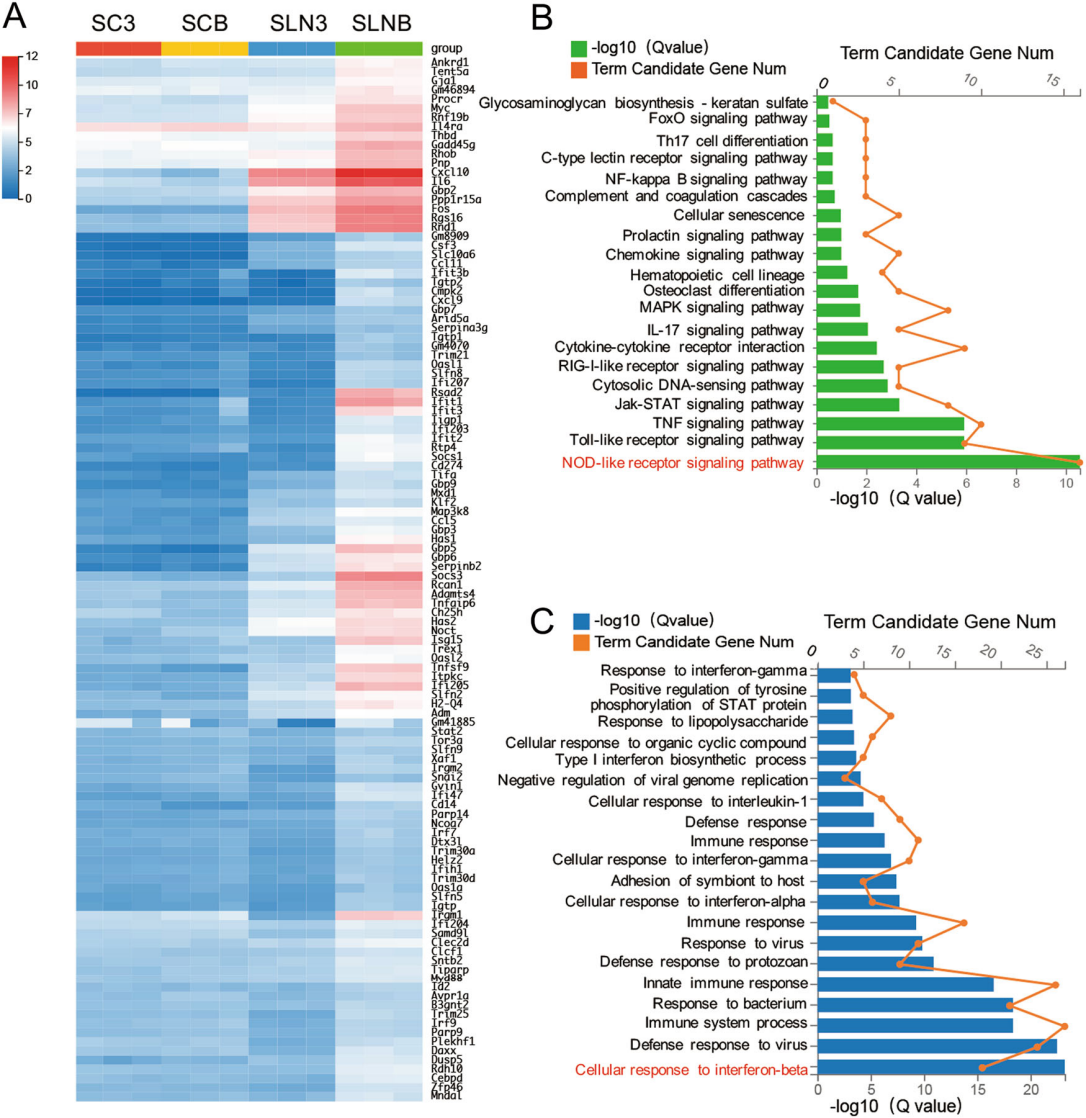
SLNB treatment modulates ASC’s transcriptome and triggers ASCs’ responses to interferon. LPS-primed ASCs were treated with SC3, SLN3, SCB, and SLNB respectively for 2 h. **a** RNA-seq heat map for ASCs exposed to SC3, SLN3, SCB, and SLNB respectively (*n* = 3). The heat map showed 114 significantly up-regulated genes (*q* < 0.001) in SLNB-treated ASCs compared to SCB-treated and SLN3-treated ASCs. Red (12) to blue (0) were ranked by values of log2(value of gene expression+1). **b** The significant KEGG pathways (*q* < 0.05) of the genes in **a**. **c** Gene Ontology analysis of the genes in **a** showing the biological process for SLNB treatment (*q* < 0.05). ASCs adipose tissue-derived mesenchymal stem cells, SC3 supernatant collected from 3T3 cells, SLN3 supernatant collected from LPS/NIG-treated 3T3 cells, SCB supernatant collected from BMDMs, SLNB supernatant collected from LPS/NIG-treated BMDMs, KEGG Kyoto Encyclopedia of Genes and Genomes, Num number.

We used RNA-seq to analyze the gene changes in SC3, SLN3, SCB, and SLNB-treated ASCs. 2390 gene expression levels in SLNB-treated ASCs were different from SCB-treated ASCs; 382 gene expression levels were different compared with SLN3-treated ASCs. Using heatmap, we listed 114 genes upregulated in SLNB-treated ASCs compared with the other groups (Fig. 5a). To identify the associated biological functions, the 114 differentially expressed genes (DEGs) were subjected to the KEGG pathways analyses. The KEGG pathway functional enrichment results (Fig. 5b) displayed 20 significant pathways (*q* < 0.05), among which NOD-like receptor signaling pathway, toll-like receptor signaling pathway, and TNF signaling pathway ranked at top 3. These findings indicated that mediators from LPS/NIG-triggered macrophages facilitate inflammasome expression in ASCs. Furthermore, the associated DEGs listed in the heatmap were subjected to GO analysis. We found that interferon related genes were noticeably upregulated in SLNB-treated ASCs. Among these genes, 18 were regulated by IFNβ, 10 by IFNγ, and 6 by IFNα (Fig. 5c).

### IFNβ expressed by LPS/NIG-stimulated BMDMs promotes ASCs pyroptosis

**Fig. 6:**
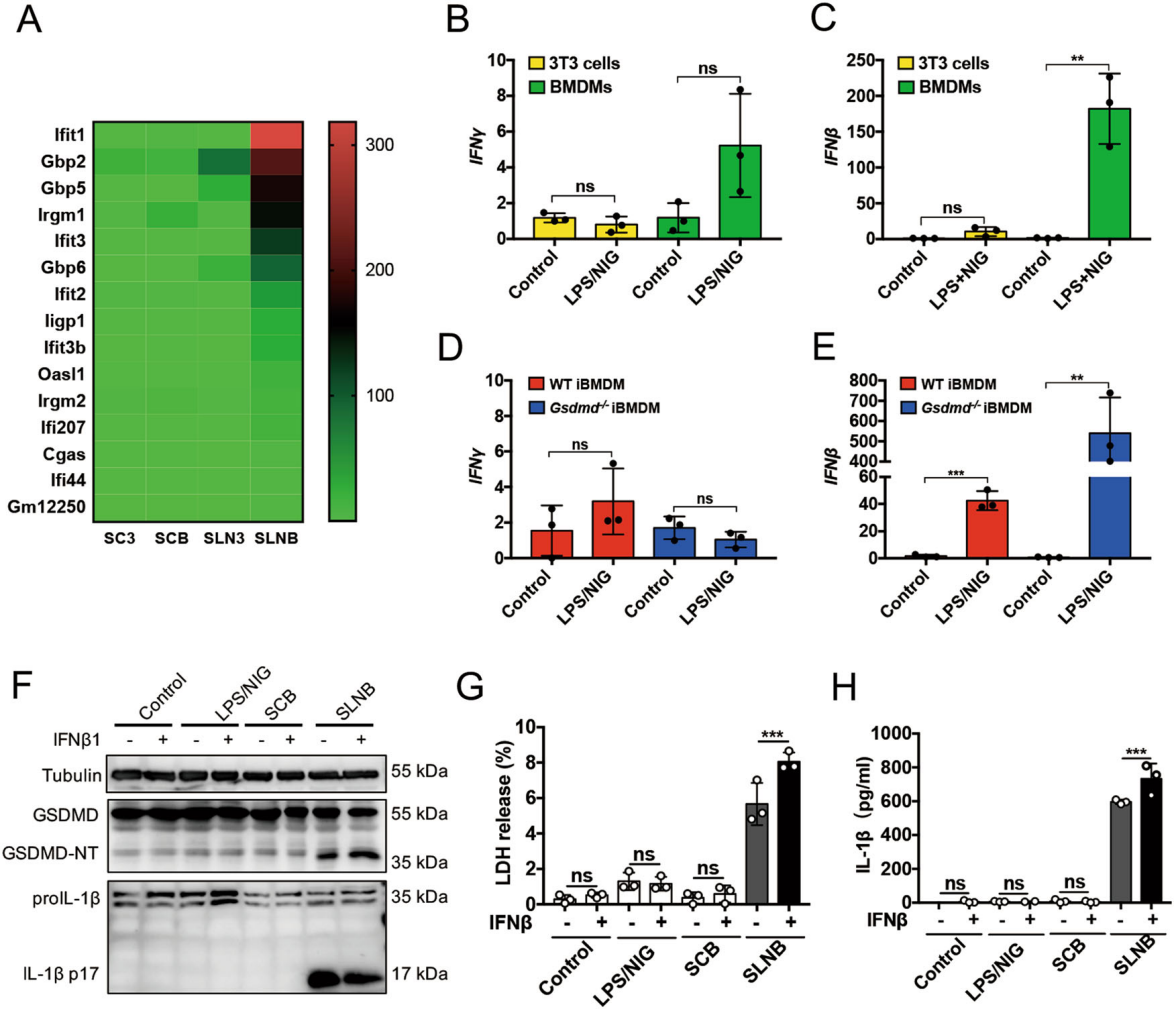
IFNβ expression increases in LPS/NIG-stimulated macrophages and IFNβ promotes pyroptosis of ASCs. **a** Heat map from RNA-seq analysis for ASCs exposed to SC3, SLN3, SCB, and SLNB respectively (*n* = 3). The heat map showed the expression level of 15 IFN-stimulated genes significantly up-regulated in SLNB-treated ASCs compared to SLN3-treated ASCs. Red (highest) to green (lowest) represent the values of gene expression level. **b**–**e** 3T3 cells, BMDMs, WT iBMDM, and *Gsdmd*^*−/−*^ iBMDM were incubating in LPS (1 μg/ml) for 5 h before being challenging with nigericin (10 μM) for 1 h. Then Cells were collected and evaluated for expression of *IFNβ* and I*FNγ* at mRNA levels by RT-PCR. Values were showed as the relative ratio of mRNA of IFNβ or IFN*γ* to mRNA of GAPDH. *n* = 3. **f**–**h** ASCs were pretreated with IFNβ (0.4 ng/ml) and LPS (0.5 μg/ml) for 4 h before being stimulated with LPS/NIG, SCB, or SLNB for 2 h. And then collected ASCs for immunoblot analysis (out of at least three performed) of GSDMD and IL-1β (**e**) or replaced with fresh medium for another 2 h. Harvest the medium for detecting LDH (**f**) and mature IL-1β (**g**) released by ASCs. *n* = 3. The statistical significance of the differences between various treatments was determined by a two-tailed *t* test for two groups or one-way analysis for three or more groups. Data are shown as mean ± SD. ns: *p* > 0.05, *p < 0.05, **p < 0.01, ***p < 0.001, ****p < 0.0001. IFN interferon, LPS lipopolysaccharide, NIG nigericin, ASCs adipose tissue-derived mesenchymal stem cells, BMDMs bone marrow-derived macrophages, iBMDMs immortalized bone marrow-derived macrophages, WT wildtype, GSDMD gasdermin D, GSDMD-NT N-terminal gasdermin D, SCB supernatant collected from BMDMs, SLNB supernatant collected from LPS/NIG-treated BMDMs, RT-PCR real-time reverse transcriptase-polymerase chain reaction, LDH lactate dehydrogenase.

Figure 6a showed that SLNB treatment significantly upregulated 15 IFN-stimulated genes of GTPase, DNA and RNA sensors in ASCs, compared to SLN3-treated ASCs. Studies have shown that some of these IFN-stimulated genes can regulate cell necrosis, especially pyroptosis^19,20^. Using qPCR analyses, we found that LPS/NIG stimulation induced a robust increase in *Ifnβ* mRNA in BMDMs (Fig. 6b, c). The *Ifnβ* expression was upregulated in both LPS/NIG-stimulated *Gsdmd*^−/−^ iBMDMs and LPS/NIG-stimulated WT iBMDMs (Fig. 6d, e). We proposed IFNβ expressed by LPS/NIG-stimulated BMDM might facilitate ASC pyroptosis. In LPS/NIG-treated ASCs, adding recombinant IFNβ to the culture system did not induce ACS pyroptosis. In SLNB-treated ASCs, combination with recombinant IFNβ enhanced cleavage of GSDMD-NT, elevated LDH release and IL-1β in the supernatant (Fig. 6f–h). These findings indicated that macrophage-released IFNβ is an enhancer for ASC pyroptosis during infection and inflammation.

### Anti-bacterial capacity is reduced in pyroptotic ASCs

**Fig. 7:**
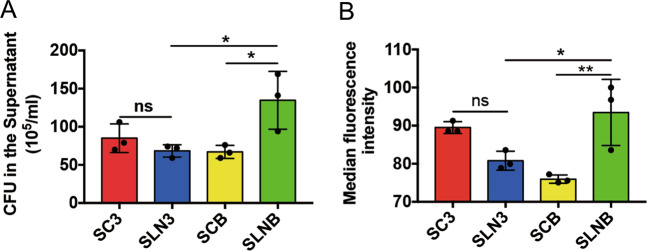
Pyroptosis reduces the antibacterial ability of ASCs. LPS-primed ASCs were incubated with SC3, SLN3, SCB, and SLNB respectively for 2 h, followed by infected with GFP-PAO1 at MOI of 1 for an additional 2 h. *n* = 3. **a** CFU counts were performed to determine the number of viable *P. aeruginosa* in the supernatants. **b** The intracellular GFP-fluorescence of ASCs was measured by a FACScan flow cytometer. The median fluorescence intensity represents the intracellular GFP-PAO1. *n* = 3. Statistical significance was determined using one-way analysis of variance. Data are shown as mean ± SD. ns: *p* > 0.05, *p < 0.05, **p < 0.01. LPS lipopolysaccharide, ASCs adipose tissue-derived mesenchymal stem cells, SC3 control supernatant collected from 3T3 cells, SLN3 supernatant collected from LPS/NIG-treated 3T3 cells, SCB control supernatant collected from BMDMs, SLNB supernatant collected from LPS/NIG-treated BMDMs, CFU colony-forming units, MOI multiplicities of infection.

Previous studies have shown that MSCs were capable of inhibiting bacteria growth by secreting antibacterial factors^21–23^, but it was unknown whether pyroptosis impaired anti-bacterial capacity in ASCs. We applied SLNB to LPS-primed ASCs to induce pyroptosis and used SC3, SLN3, or SCB-treated ASCs as controls. After the above treatment, the treated ASCs were infected with *P. aeruginosa* (MOI = 1). And then CFUs in the supernatant were measured by serial dilution. CFUs in the SLNB-treated ASCs group were increased compared to the other groups (Fig. 7a), suggesting that pyroptotic ASCs have an impaired extracellular antibacterial ability.

To assess whether intracellular bacteria killing-ability was affected in pyroptotic ASCs, we infected SLNB-treated ASCs with GFP-PAO1 and used SC3, SLN3, or SCB-treated ASCs as controls. The cells were collected for evaluating intracellular bacteria fluorescence intensity by flow cytometry. Median fluorescence intensity (MFI) in the SLNB-stimulated GFP-PAO1-infected ASCs was higher compared to the SLN3 or SCB-stimulated GFP-PAO1-infected ASCs (Fig. 7b), indicating that pyroptotic ASCs have an impaired intracellular antibacterial ability.

## Discussion

Host inflammatory microenvironments have a profound impact on MSCs during cell-based therapies^24,25^. MSCs might undergo autophagy and apoptosis in highly inflammatory environments. Autophagy and apoptosis could change the immunomodulatory property of MSCs^5–7^. Pyroptosis is a form of programmed lytic cell death mediated by inflammatory caspases activated by cytosolic microbial signals mainly described in macrophage/dendritic cells^9^. In the present study, we used LPS/NIG-stimulated macrophage media (SLNB) to challenge LPS-primed ASCs and found that ASCs underwent pyroptosis. Thus, for the first time, we report that ASCs can develop pyroptosis under the stimulation with mediators from pyroptotic macrophages.

ASCs are the popularly used MSCs since they are in large quantities, easily isolated, and less ethical issues^26,27^. ASCs possess antibacterial, immunomodulatory, and tissue-regenerative capacities which make ASCs accessible to clinical trials for various diseases^28^. Mouse’ ASCs isolated from the inguinal area were well documented and their identification and properties of stem cells have been confirmed^13,29,30^. Thus, the ASCs isolated from the inguinal area of mice were selected in this study.

To study ASC pyroptosis, we approached diverse inflammasome agonists: LPS, flagellin, dsDNA, and nigericin to activate caspase-11, NLRC4, AIM2, or NLRP3 inflammasomes respectively^8^. By immunoblotting analyses, we confirm that ASCs expressed caspase-1, caspase-11, GSDMD, and IL-1β, which are required for the induction of pyroptosis. However, we found that ASCs do not undergo pyroptosis no matter whether the ASCs were incubated or transfected with LPS, flagellin, dsDNA, or nigericin or LPS combined with flagellin, dsDNA, or nigericin. Resistance to LPS, flagellin, dsDNA, or nigericin-induced pyroptosis in ASCs might be conducive for ASCs to perform a better function during cell-based therapy.

In the inflammatory microenvironments, ASCs are inevitably exposed to varieties of damage associated molecular patterns (DAMPs) besides PAMPs^31^. Macrophages, as the first line of immune defense, could secrete large amounts of proinflammatory cytokines and undergo pyroptosis if challenged with PAMPs^32^. We collected the culture medium from LPS/NIG triggered pyroptotic macrophages and apply the collected media to incubate ASCs. Interestingly, we found that SLNB induced cleavage of GSDMD and proIL-1β in ASCs. To demonstrate that SLNB triggers ASC pyroptosis, we replaced SLNB with fresh media after SLNB incubating ASCs. We were still able to measure high levels of LDH and IL-1β in the supernatant of fresh media-incubated ASCs. These findings indicate that SLNB elicit pyroptosis of ASCs, and once the cleavage of GSDMD and proIL-1β in ASC are initiated, the cascade could be propagated. For the first time, we have demonstrated that ASCs can undergo pyroptosis, which requiring mediators from LPS/NIG-stimulated macrophages.

However, the pyroptosis in ASCs is independent of their own caspase-1/11, suggesting that macrophage-derived activated forms of caspase-1/11 might be key mediators that could induce ASC pyroptosis. To confirm this, we applied LPS/NIG-stimulated *Caspase-1/11*^−/−^ or *Gsdmd*^−/−^ macrophage-media to ASCs. The results show that SLNB from *Caspase-1/11*^−/−^ or *Gsdmd*^−/−^ macrophage could not induce ASC pyroptosis. It is generally accepted that pyroptosis relied on caspase-1 or caspase-11 mediated cleavage of GSDMD^8^. Some studies have shown that monocyte-derived microvesicles containing activated caspase-1 and GSDMD could induce pulmonary vascular endothelial cell death^33,34^. Therefore, we speculate that macrophage-derived activated caspase-1 and 11 forms are required for cleaving ASC GSDMD and proIL-1β. Now, we lack tools to neutralize or inhibit activated caspase-1 and 11 forms in the SLNB. We are trying to develop a way to remove activated forms of caspase-1 and 11 from the SLNB. Further studies are required to confirm this speculation.

In this study, we have to note that compared to macrophages, the proportion of pyroptotic ASCs is relatively low since only a minority of ASCs (~10%) undergo pyroptosis. We speculate that the ASCs uptake the activated caspase-1 and 11 forms present in the SLNB. These activated caspase-1 and 11 forms cleave GSDMD and proIL-1β in the ASCs not as on a large scale as in the macrophages. It is also reported compared to the macrophages, neutrophils, dendritic cells, and human monocytes have less capacity to release active IL-1β with minor cell death^35–37^.

Besides active caspase-1/11 forms presenting in the media of LPS/NIG-stimulated macrophages, we found that IFNβ was also expressed in LPS/NIG-stimulated macrophages. RNA-seq data have demonstrated that the expression of IFN-stimulated genes was markedly increased in the SLNB-treated ASCs. Among these IFN-stimulated genes, there are 15 IFN-stimulated genes of GTPase, DNA and RNA sensors, which have been reported that their gene products might facilitate sensing microbial components. For example, Gm12250, GBP2 and 5 could liberate bacterial ligands for being sensed by inflammasomes and consequently facilitate pyroptosis^38–40^. Corresponding with these studies, we found that IFNβ could boost SLNB-mediated ASC pyroptosis. Interestingly, although the deletion of *Gsdmd* enhances the IFNβ expression in BMDMs^41^, due to lack of active caspase-1/11, SLNB from *GSDMD*^*−/−*^ BMDMs could not induce pyroptosis of ASCs. Thus, we speculate that IFNβ is a key mediator presenting in the SLNB that could aggravate ASC pyroptosis but not execute ASC pyroptosis.

We have mentioned that MSCs could attenuate infection by secreting antibacterial factors (lipocalin, LL37, β-defensin-2) and phagocytosing bacteria during infection^42^. In this study, the pyroptotic ASCs have a reduced bactericidal ability to *P. Aeruginosa* extracellularly and intracellularly. The clinical relevance of this phenomenon is worthy of investigation in clinical setting.

In conclusion, ASCs undergo pyroptosis in response to the media collected from LPS/NIG-stimulated macrophages. ASCs might use activated forms of caspase-1/11 and IFN-β from macrophages to initiate and amplify their own pyroptosis. The pyroptotic ASCs are less potent in antibacterial capacity. These findings will provide us new insight into understanding the fate and function of MSCs when the MSCs are administrated into the inflammatory microenvironments.

## Supplementary information

Supplementary figure legends

supplemental figure 1

Supplemental figure 2
