# Supplementary material for: Induction of ASC pyroptosis requires gasdermin D or caspase-1/11-dependent mediators and IFNβ from pyroptotic macrophages
Source: Cell Death Dis. 2020 Jun 18;11(6):470. doi: 10.1038/s41419-020-2664-0 (PMC7303158; doi:10.1038/s41419-020-2664-0)
Supplement: Supplementary file 1 — Supplementary figure legends [file 41419_2020_2664_MOESM1_ESM.docx]

**Supplementary Figure Legends**

**Supplemental Figure 1**

**LPS/nigericin irritate pyroptosis on BMDMs but not on ASCs or 3T3 cells, which can be inhibited by VX765. (A-C):** ASCs, 3T3 cells, and BMDMs were treated with LPS/nigericin. **(D, E)** BMDMs were pretreated with VX765 before being treated with LPS and nigericin. A representative Western blot is shown out of 3 performed.

**Supplemental Figure 2**

***Caspase-1/11^-/-^* and *Gsdmd^-/-^* macrophages cannot develop pyroptosis under stimulation of LPS/NIG.** WT BMDMs, *caspase-1/11^-/-^* BMDMs, WT iBMDMs, and *Gsdmd^-/-^* iBMDMs were treated with LPS or nigericin. Pyroptosis was detected by immunoblot analysis **(A, D),** ELISA detection **(B, E)** and LDH release **(C, F)** insupernatants. N=3. A representative Western blot is shown out of 3 performed.
