# Supplementary figures and images for: Induction of ASC pyroptosis requires gasdermin D or caspase-1/11-dependent mediators and IFNβ from pyroptotic macrophages
Source: Cell Death Dis. 2020 Jun 18;11(6):470. doi: 10.1038/s41419-020-2664-0 (PMC7303158; doi:10.1038/s41419-020-2664-0)

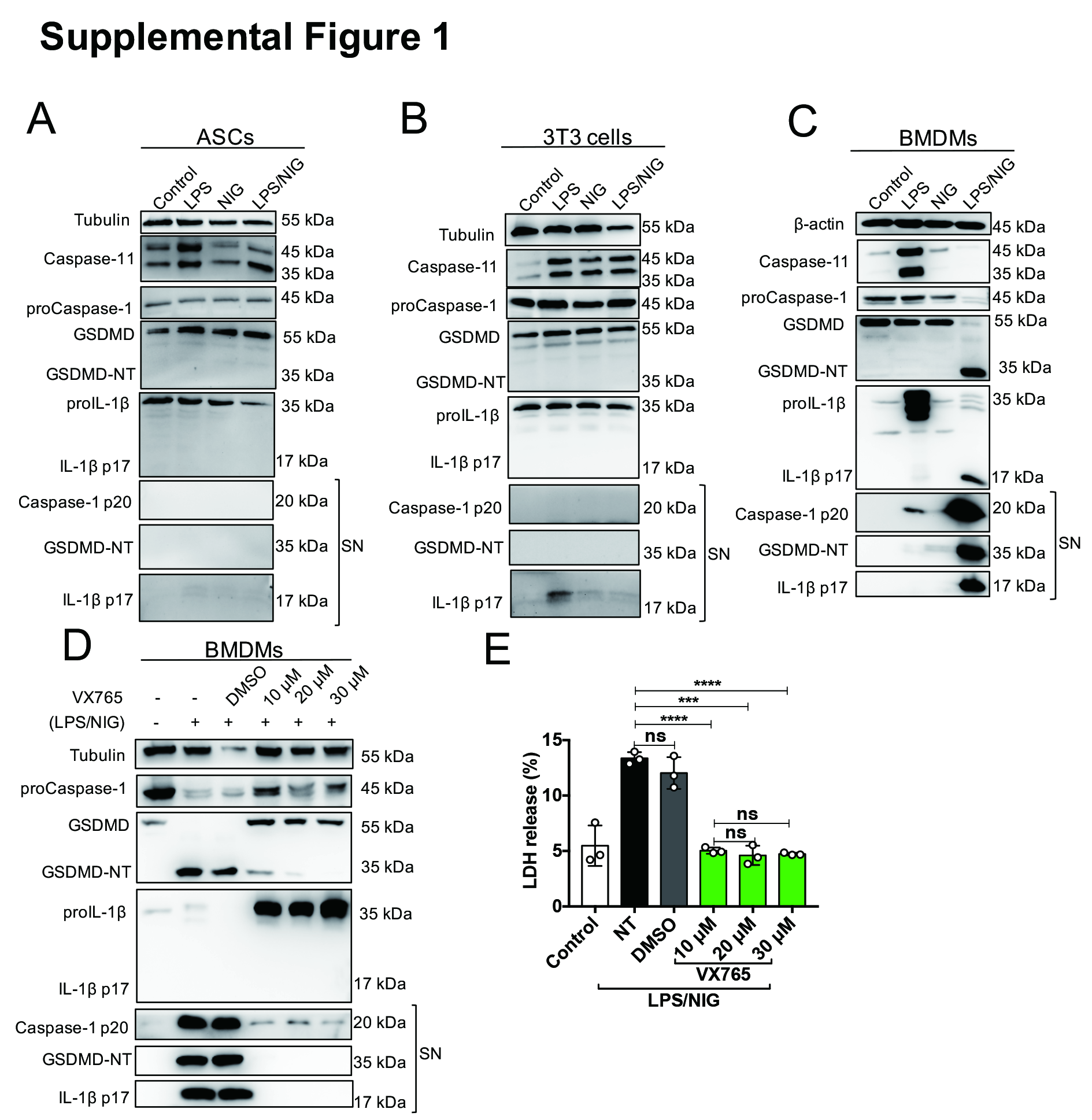

Supplement: Supplementary file 2 — supplemental figure 1 [file 41419_2020_2664_MOESM2_ESM.tif]

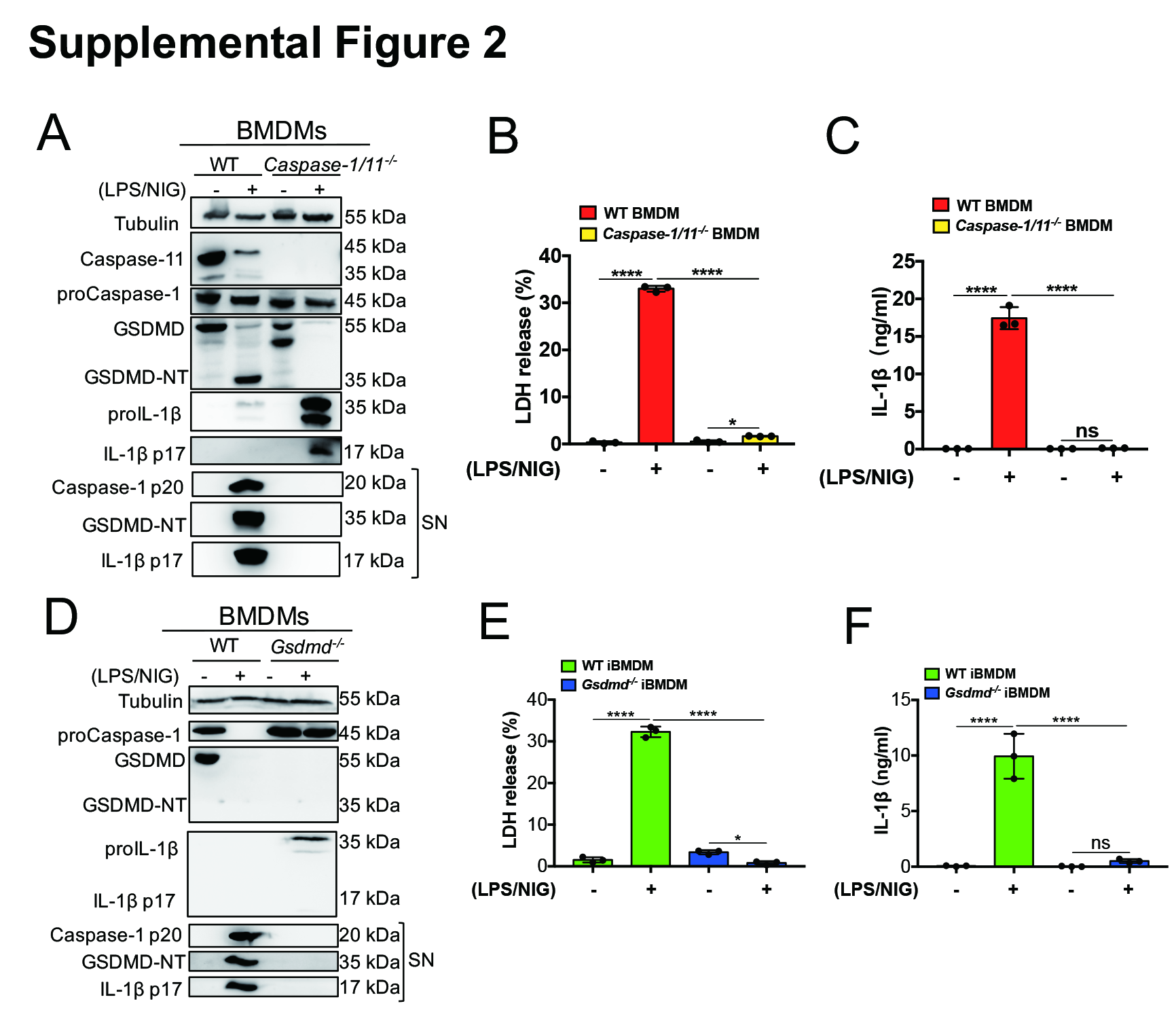

Supplement: Supplementary file 3 — Supplemental figure 2 [file 41419_2020_2664_MOESM3_ESM.tif]
